# Similarities and Differences between Clavicular Bacterial Osteomyelitis and Nonbacterial Osteitis: Comparisons of 327 Reported Cases

**DOI:** 10.1155/2021/4634505

**Published:** 2021-01-26

**Authors:** Nan Jiang, Ping Zhang, Wei-ran Hu, Zi-long Yao, Bin Yu

**Affiliations:** ^1^Division of Orthopaedics & Traumatology, Department of Orthopaedics, Nanfang Hospital, Southern Medical University, Guangzhou 510515, China; ^2^Guangdong Provincial Key Laboratory of Bone & Cartilage Regenerative Medicine, Nanfang Hospital, Southern Medical University, Guangzhou 510515, China; ^3^Department of Orthopaedics, Henan Provincial People's Hospital, Zhengzhou 450003, China

## Abstract

**Background:**

Currently, both clavicular bacterial osteomyelitis (BO) and nonbacterial osteitis (NBO) remain not well understood owing to their much lower incidences. This study is aimed at summarizing similarities and differences between clavicular BO and NBO based on comparisons of literature-reported cases.

**Methods:**

We searched the PubMed and Embase databases to identify English published literature between January 1^st^, 1980, and December 31^st^, 2018. Inclusion criteria were studies evaluating clinical features, diagnosis, and treatment of clavicular BO and NBO, with eligible data for synthesis analysis.

**Results:**

Altogether, 129 studies with 327 patients were included. Compared with BO, clavicular NBO favored females (*P* < 0.001) and age below 20 years (*P* < 0.001) and mostly presented in a chronic phase (disease term exceeding 2 months) (*P* < 0.001). Although local pain and swelling were the top two symptoms for both disorders, fever, erythema, and a sinus tract were more frequently found in BO patients (*P* < 0.01). Although they both favored the medial side, lesions in the clavicular lateral side mostly occurred in BO patients (*P* = 0.002). However, no significant differences were identified regarding the serological levels of white blood cell count (*P* = 0.06), erythrocyte sedimentation rate (*P* = 0.27), or C-reactive protein (*P* = 0.33) between BO and NBO patients before therapy. Overall, the BO patients achieved a statistically higher cure rate than that of the NBO patients (*P* = 0.018).

**Conclusions:**

Females, age below 20 years, and a long duration of clavicular pain and swelling may imply NBO. While the occurrence of a sinus tract and lesions in the lateral side may be clues of BO, inflammatory biomarkers revealed limited values for differential diagnosis. BO patients could achieve a better efficacy than the NBO patients based on current evidence.

## 1. Introduction

Clavicular inflammatory disorders can be classified as bacterial osteomyelitis (BO) and nonbacterial osteitis (NBO) according to different etiologies. Clavicular BO, caused by pathogen invasions via contiguous, perioperative, and hematogenous routes [1–4], contains infectious osteomyelitis (OM) and septic arthritis (SA) of the joints surrounding the clavicle. Infectious OM, which refers to inflammation-related osteonecrosis, osteolysis, and/or new bone formation with or without surrounding soft tissue involvement [5], primarily affects bones in the lower extremity, such as the tibia, femur, and metatarsal bones [6, 7], while SA usually leads to joint cartilage damage, host inflammation, and tissue ischemia following pathogen invasion, with the knee joint as the most frequently involved site [8]. However, clavicular BO remains seldom reported because of its much lower prevalence [9].

NBO, different from BO, acted as an autoinflammatory bone disorder not related to infectious disease, which is characterized by activation of the innate immune system in the absence of high-titer autoantibodies without the involvement of the autoreactive lymphocytes (at least initially) [10]. In 1972, Giedion et al. [11] first described this disorder, known as chronic recurrent multifocal osteomyelitis (CRMO). Nowadays, aside from CRMO, NBO has become a broad concept disorder, including synovitis-acne-pustulosis-hyperostosis-osteitis (SAPHO) syndrome, condensing osteitis, and even hyperostosis [12]. Despite its wide concept, up till now, the number of studies reporting clavicular NBO is also quite limited, which likewise associates with the much lower prevalence [13].

Due to their much lower prevalences, currently, both clavicular BO and NBO are still not well understood. In a previous review [14], we summarized clinical characteristics and treatment strategy of clavicular OM based on literature-reported cases; however, as mentioned in the study, both BO and NBO cases were included as a whole for analysis, resulting in a higher risk of heterogeneity. Although the two disorders share similarities, they also possess differences.

It is very important to distinguish clavicular BO and NBO patients as being able to do this can help clinicians avoid unnecessary diagnostic procedures, antibiotic therapies, and even surgeries. However, to the best of our knowledge, up till now, only one German study [9], based on a nationwide survey, reported similarities and differences between BO and NBO in pediatric patients. However, this study analyzed the whole-body bones. Currently, there still lacks such an analysis to differentiate BO and NBO of the clavicle. Therefore, this study is aimed at summarizing similarities and differences of clinical characteristics, diagnosis, and treatment between clavicular BO and NBO.

## 2. Methods

### 2.1. Literature Search, Definition, and Inclusion and Exclusion Criteria

A systematic literature search was conducted in the PubMed and Embase databases by two independent authors, to identify English studies reporting clavicular BO and NBO, published between January 1^st^, 1980, and December 31^st^, 2018. Terms used for the search were “clavicle,” “clavicular,” “osteomyelitis,” “osteitis,” and “hyperostosis.” Clavicular BO refers to infectious OM and septic arthritis of the surrounding joints, such as acromioclavicular and sternoclavicular joints, while clavicular NBO includes CRMO, SAPHO syndrome, condensing osteitis, and hyperostosis. The disease phase is defined as the duration from the onset of symptoms to clinical diagnosis, with the acute phase shorter than 2 months and the chronic phase longer than 2 months. Inclusion criteria of the present study were human studies evaluating clinical characteristics, diagnosis, and treatment of either clavicular BO or NBO or both, with eligible data (described in Section 2.3) for synthesis analysis. Case report/series, letter to the editor article, and conference paper were also screened only if they satisfied the inclusion criteria. Exclusion criteria included non-English published literature and studies without clavicular BO or NBO or without available data for pooled analysis. In addition, other study types (e.g., systematic reviews and meta-analyses) and research fields (e.g., radiology and flap surgery) were also excluded.

### 2.2. Study Identification and Data Extraction

Two authors independently screened titles of the identified studies initially, and unrelated ones were excluded. Subsequently, abstracts that were potentially relevant to the topic were reviewed. If studies appeared to be potentially applicable, full texts were reviewed for further evaluation of whether the studies satisfied all the inclusion criteria. Three authors participated in the extraction of the effective data from all eligible studies independently. Disagreement about eligibility and harvested data was resolved by discussion, and if necessary, the corresponding author's opinion was consulted to make the final decision.

### 2.3. Data Collection

Eligible data collected from included studies were the disorder type; sex; age and disease phase; clinical symptom and duration; pathogen culture results of the BO patients; body side and site; serological levels of white blood cell count (WBC), erythrocyte sedimentation rate (ESR), and C-reactive protein (CRP) before therapy; treatment strategy; and efficacy.

### 2.4. Statistical Analysis

Statistical analysis was performed using the Statistical Package for the Social Sciences (SPSS) 17.0 software (SPSS Inc., Chicago, IL, USA). Distributions of the continuous variables were evaluated for normality using the Kolmogorov-Smirnov test. Then, the data were presented as the mean ± standard deviation (SD) or median with interquartile range (IQR) depending on data distribution. For normally distributed data, Student's *t*-test was used to compare differences between the two groups. Otherwise, the Mann-Whitney *U* test was applied. Dichotomous variables were expressed as percentages with events and totals. The chi-squared test was used to compare differences of rates between the two groups. A statistically significant difference was defined as a *P* value ≤ 0.05.

## 3. Results

### 3.1. Study Identification and Constitutions of Clavicular BO and NBO

**Figure 1:**
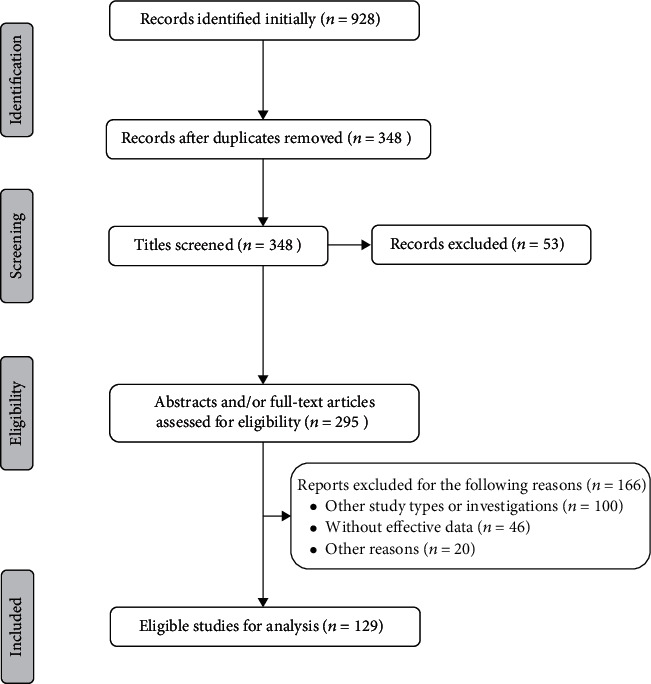
Flow diagram of the included studies in this review.

Altogether, 928 reports were found initially; after removing the duplicates and applying the inclusion criteria, we finally included 129 studies of 327 patients [1–4, 12, 15–138] (Figure 1). Of the eligible 327 cases, 171 patients were classified as BO, with 39 caused by *Mycobacterium tuberculosis*. Among the 156 NBO cases, 66 were reported as condensing osteitis, 61 as CRMO, 21 as SAPHO syndrome, and the remaining 8 as sternocostoclavicular hyperostosis.

### 3.2. Clinical Characteristics of Clavicular BO vs. NBO

#### 3.2.1. Sex Ratio and Age at Diagnosis

**Figure 2:**
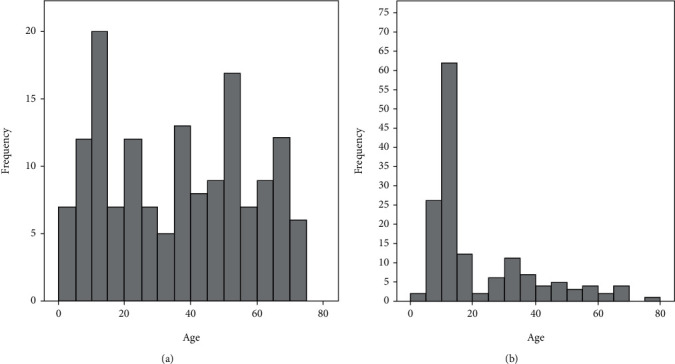
Age distributions of the included clavicular BO (a) and NBO (b) patients.

As shown in Table 1, a significant difference was identified regarding the female-to-male ratio between clavicular BO and NBO (1.01 vs. 3.08, *P* < 0.001), suggesting that clavicular NBO favored females. Additionally, the median age of NBO patients at diagnosis was significantly younger than that of the BO patients (13 years vs. 36 years, *P* < 0.001). The percentage of the NBO patients younger than 20 years at diagnosis was significantly higher than that of the BO patients (67.5% vs. 30.5%, *P* < 0.001), demonstrating that clavicular NBO was predominant in young people (Figure 2).

#### 3.2.2. Disease Phase Ratio and Symptom Duration

Compared with the BO patients, the majority of the NBO patients were presented in a chronic phase (disease term ≥ 2 months) at diagnosis (91.4% vs. 55.1%, *P* < 0.001), with a significantly higher chronic-to-acute ratio of NBO than that of BO (10.6 vs. 1.2, *P* < 0.001). In addition, the median symptom duration of the NBO patients was twice longer than that of the BO patients (6 months vs. 3 months, *P* < 0.001) (Table 1).

#### 3.2.3. Clinical Symptoms

Although the top two symptoms of both BO and NBO patients were local pain and swelling, the ratios of the two symptoms in the NBO patients were significantly higher than those of the BO patients (*P* < 0.005), while fever, erythema, and sinus tract were more frequently found in the BO patients (*P* < 0.01). However, no statistical difference was identified regarding the proportion of tenderness between BO and NBO patients (*P* = 0.502) (Table 1).

#### 3.2.4. Pathogen Culture Outcomes of the BO Patients Included

**Figure 3:**
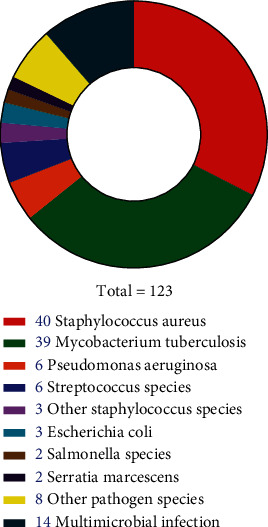
Distributions of pathogen culture outcomes of the BO patients included.

The total positive rate of culture was 84.27% (123/146), with 109 and 14 patients having a monomicrobial and a multimicrobial infection, respectively.  Figure 3 depicts the distributions of bacterial species, with *Staphylococcus aureus* (36.70%) as the most frequent type, followed by *Mycobacterium tuberculosis* (35.78%), *Pseudomonas aeruginosa* (5.50%), and *Streptococcus species* (5.50%), respectively.

#### 3.2.5. Involved Side and Site Distributions

Both BO and NBO favored the right body side, with no statistical differences of side distributions between them. However, the percentage of the multifocal lesions in the clavicle of the BO patients was significantly higher than that of the NBO patients (17.9% vs. 7.5%, *P* = 0.018). As for the patients with a unifocal lesion, both BO and NBO favored the medial side. However, the percentage of lesions located on the medial side was statistically higher in the NBO patients (89.8% vs. 79.8%, *P* = 0.044). Conversely, BO favored the lateral side more than NBO (11.8% vs. 1.0%, *P* = 0.002).

### 3.3. Diagnosis of Clavicular BO vs. NBO

#### 3.3.1. Serological Levels of Inflammatory Biomarkers before Therapy

As revealed in Table 2, no statistical differences were found regarding the serological levels of WBC (*P* = 0.064), ESR (*P* = 0.272), or CRP (*P* = 0.330) between BO and NBO patients prior to therapy. In the stratified analyses by disease phase, no statistical differences were identified for the levels of such biomarkers between the two disorders, neither in an acute phase nor in a chronic phase.

### 3.4. Treatment and Efficacy of Clavicular BO vs. NBO

#### 3.4.1. Cure Rate of Clavicular BO vs. NBO

Although outcomes revealed no statistical differences regarding the cure rates following surgery or nonsurgery between the BO and NBO patients, the overall cure rate of the BO patients was significantly higher than the NBO patients (89.5% vs. 76.8%, *P* = 0.018), implying probably better efficacy of the BO patients (Table 3).

#### 3.4.2. Cure Rate of Surgery vs. Nonsurgery for Clavicular BO and NBO

As shown in Table 3, no significant differences were observed in the cure rates after surgical and nonsurgical interventions, neither in BO patients (92.5% vs. 86.0%, *P* = 0.234) nor in NBO patients (81.8% vs. 75.9%, *P* = 0.668).

## 4. Discussion

Outcomes of this study demonstrated that both similarities and differences have been found between clavicular BO and NBO. Based on the synthesis analysis, we concluded that females, people younger than 20 years, and long duration (over 2 months) of clavicular pain and swelling may imply NBO, while a local sinus tract and lesions in the lateral side of the clavicle may be clues of BO. However, serological levels of WBC, ESR, and CRP had limited values for differential diagnosis. Despite treatment strategies, BO patients might achieve better efficacy than NBO patients. Our findings can be summarized with the following primary three aspects.

First, we summarized similarities and differences of clinical characteristics between clavicular BO and NBO. Initially, we noticed that compared with BO, NBO favored females and people below 20 years, which were in accordance with previous studies [9, 13, 139–141]. In addition, we also noted that the proportion of NBO patients in a chronic phase at diagnosis was significantly higher than that of the BO patients, implying that NBO may be subtler than BO in most cases. Therefore, it is reasonable to understand why the median symptom duration of the NBO patients was longer than BO patients. In addition to the disease type, symptom duration is also influenced by other factors, such as its severity, host immune status, and medical interventions.

Clinical symptom is an important clue for differentiating BO and NBO. Although local pain and swelling were the most frequently reported symptoms for both BO and NBO, it is unsuitable to be regarded as distinguishing indicators. Likewise, tenderness is also not helpful as both of their percentages were low. However, ratios of fever, erythema, and a sinus tract in the BO patients were significantly higher than those in the NBO patients. Of the above three symptoms, we believe a sinus tract was mostly clinically significant, as a sinus tract with communication to the bone or implant is one of the confirmatory criteria for diagnosis of bacterial osseous infection [142]. It is interesting that among the included studies, one case reported as SAPHO syndrome was also with a discharging sinus tract, with a pathogen culture outcome of *P. acnes* [41]. Therefore, a sinus tract implies BO in most cases but cannot completely rule out NBO, especially for the culture outcome of *P. acnes.* With regard to pathogen culture results of the BO patients, outcomes revealed that *Staphylococcus aureus* and *Pseudomonas aeruginosa* were more frequently detected, which was in accordance with our previous studies regarding extremity infectious OM [6, 143]. Aside from the two pathogens, *Mycobacterium tuberculosis* was also frequently found in BO patients; considering particularity of this bacteria, *Mycobacterium tuberculosis*-related clavicular BO should be further investigated.

Lesion location may be another important clue for distinguishing BO and NBO. Here, we failed to find any statistical difference of body side distribution between them, both of which favored the right side. However, the proportion of the multifocal lesions in the clavicle in BO patients was significantly higher than that of the NBO patients. Additionally, we noticed that the lesions were mostly located in the medial side in both BO and NBO patients, though a slightly higher percentage in the NBO patients, which should not be regarded as an effective indicator to differentiate the two diseases. In contrast, the percentage of the lesions on the lateral side of the BO patients was much higher than that in the NBO patients, which may be a clue of BO.

Second, we investigated potential values of serological WBC, ESR, and CRP for differential diagnosis of BO and NBO. Unfortunately, we failed to observe any significant differences between BO and NBO, neither in an acute phase nor in a chronic phase. Therefore, serological levels of WBC, ESR, or CRP may be inappropriate to distinguish the two disorders. Similarly, aside from the disease phase, many other factors may also affect serological levels of these biomarkers, such as bacteria species and virulence, host status, lesion number, and previous treatment. In addition to the above factors, the limited sample size, especially for the patients in the acute phase, may also influence the outcomes. Here, we did not analyze the potential roles of imaging tests in the assisted diagnosis of BO and NBO because of the unavailability for pooling of such data. However, imaging tests, including conventional radiography, computed tomography (CT), magnetic resonance imaging (MRI), bone scintigraphy (BS), and whole-body MRI (WB-MRI), are another effective way to differentiate the two disorders in some selected patients [9].

Third, we compared the cure rates after surgical and nonsurgical interventions between BO and NBO. On the one hand, the overall cure rate of the BO patients (90%) was significantly higher than that of the NBO patients (77%), demonstrating probably better efficacy of BO patients. However, such a difference between BO and NBO may be just statistically different, as they both revealed fairly high response rates. Considering the very different nature of BO and NBO, the follow-up time is also an important factor that may affect the outcomes. Additionally, such a difference also should be interpreted with caution as currently, management of clavicular BO and NBO remains primarily empirical. Indications for surgery or nonsurgery still are a hot debate. Likewise, surgery or nonsurgery just to be one issue, disorder type, clinicians' experiences, patients' compliances, and surgical or nonsurgical strategies may also affect clinical efficacy. On the other hand, although surgically treated BO and NBO patients had slightly higher cure rates than their respective conservative treatment, no significant differences were found between surgery and nonsurgery, which is thought-provoking. Whether surgery remains essential and indications of surgery for both BO and NBO should be further investigated.

The present study also had limitations. Although this updated study categorized clavicular OM as BO and NBO, heterogeneity may be lower to some extent but cannot be eliminated absolutely. Although both OM and SA were included as BO, with CRMO, SAPHO syndrome, condensing osteitis, and hyperostosis recruited as NBO, comparisons between BO and NBO which are constituted of different disorder types may have biases. Therefore, in-depth or stratified analyses may be necessary. Additionally, although this study included all available cases reported between 1980 and 2018, the total number of eligible patients was still limited, especially for the subtypes of NBO. Therefore, a larger sample size is warranted to obtain more accurate conclusions in the future. Moreover, publication bias might be another limitation as many of the patients diagnosed with BO/NBO may have never been reported or published.

## 5. Conclusions

In summary, this updated synthesis analysis with 327 cases suggested that females, people younger than 20 years, and a long duration of clavicular site pain and swelling may imply NBO. While the occurrence of a sinus tract and lesion in the clavicular lateral side may be important clues of BO. Serological levels of WBC, ESR, and CRP had limited differential diagnostic values. The overall cure rate of clavicular BO patients was higher than that of NBO patients. However, the current evidence did not support the belief that surgery could bring better efficacy than nonsurgery, neither in the BO patients nor in the NBO patients.

## Figures and Tables

**Table 1:** Similarities and differences of clinical characteristics between clavicular BO and NBO.

Items	Clavicular BO	Clavicular NBO	Statistics	*P* values
Included cases	171	156	—	—
Sex ratio (female/male)	1.0 (76/75)	3.1 (114/37)	20.493	<0.001
Age at diagnosis (year)	36 (14, 53)	13 (10, 32)	﹣5.842	<0.001
Percentage of patients below 20 years	30.5 (46/151)	67.5 (102/151)	41.553	<0.001
Disease phase ratio (chronic/acute)	1.2 (76/62)	10.6 (117/11)	44.026	<0.001
Symptom duration (month)	3 (0.55, 7.0)	6 (3.0, 22.5)	﹣4.134	<0.001
Clinical symptoms	97/78/40/29/34/24	118/96/15/2/1/16	66.850	<0.001
Pain	32.1 (97/302)	47.6 (118/248)	13.672	<0.001
Swelling	25.8 (78/302)	38.7 (96/248)	10.448	0.001
Fever	13.2 (40/302)	6.0 (15/248)	7.836	0.005
Erythema	9.6 (29/302)	0.8 (2/248)	19.810	<0.001
Sinus	11.3 (34/302)	0.4 (1/248)	26.928	<0.001
Tenderness	8.0 (24/302)	6.5 (16/248)	0.452	0.502
Body side distribution (left/right/bilateral)	53/79/5	52/67/7	0.871	0.647
Left	38.7 (53/137)	41.3 (52/126)	0.183	0.669
Right	57.7 (79/137)	53.2 (67/126)	0.536	0.464
Bilateral	3.6 (5/137)	5.5 (7/126)	0.548	0.459
Multifocal lesions in clavicle	17.9 (26/145)	7.5 (8/106)	5.638	0.018
Unifocal distribution (medial/middle/lateral)	95/10/14	88/9/1	9.645	0.008
Medial (including SC joint)	79.8 (95/119)	89.8 (88/98)	4.038	0.044
Middle	8.4 (10/119)	9.2 (9/98)	0.041	0.840
Lateral (including AC joint)	11.8 (14/119)	1.0 (1/98)	9.642	0.002

BO: bacterial osteomyelitis; NBO: nonbacterial osteitis; SC: sternoclavicular; AC: acromioclavicular. Continuous variables are presented as the median with interquartile range. Dichotomous variables are expressed as percentages and events with totals.

**Table 2:** Serological levels of WBC, ESR, and CRP for differential diagnosis of clavicular BO and NBO.

Items	Clavicular BO	Clavicular NBO	Statistics	*P* values
Serological levels of inflammatory biomarkers before therapy (for overall patients)				
WBC (×10^9^/L)	9.8 (8.45, 16.25)	9.25 (6.72, 12.37)	﹣1.850	0.064
ESR (mm/1 h)	56 (35, 92)	49 (30, 77)	﹣1.098	0.272
CRP (mg/L)	20 (8.25, 63.75)	20.1 (2.5, 38.5)	﹣0.974	0.330
Serological levels of inflammatory biomarkers before therapy (for patients in acute phase at diagnosis)				
WBC (×10^9^/L)	9.77 (7.30, 17.15)	9.95 (7.35, 11.65)	﹣0.154	0.877
ESR (mm/1 h)	60 (39.5, 108)	36.5 (11.25, 67)	﹣1.004	0.315
CRP (mg/L)	20.5 (6.32, 77.3)	41.85 (25.7, 58)	﹣0.783	0.433
Serological levels of inflammatory biomarkers before therapy (for patients in chronic phase at diagnosis)				
WBC (×10^9^/L)	9.6 (8.2, 14.2)	8.27 (6.65, 11.8)	﹣1.692	0.091
ESR (mm/1 h)	55.5 (35, 78.25)	49 (30, 77)	﹣0.616	0.538
CRP (mg/L)	26 (10.4, 147)	9 (2.5, 34)	﹣1.622	0.105

BO: bacterial osteomyelitis; NBO: nonbacterial osteitis; WBC: white cell blood count; ESR: erythrocyte sedimentation rate; CRP: C-reactive protein. Continuous variables are expressed as median with interquartile range.

**Table 3:** Treatment strategy and efficacy between clavicular BO and NBO.

Treatment strategy	Clavicular BO	Clavicular NBO	Statistics	*P* values
Surgery	92.5 (62/67)	81.8 (9/11)	1.102	0.294
Debridement	91.1 (41/45)	100 (3/3)		
Partial resection	93.8 (15/16)	50 (2/4)^**#**^
Total/subtotal resection	100 (6/6)	100 (4/4)
Nonsurgery	86.0 (49/57)	75.9 (44/58)	1.897	0.168
Overall cure rate	89.5 (111/124)	76.8 (53/69)	5.604	0.018
Statistics^∗^	1.418	0.184		
*P* values^∗^	0.234	0.668		

BO: bacterial osteomyelitis; NBO: nonbacterial osteitis. Dichotomous variables are expressed as percentages and events with totals. ^#^No patients diagnosed with clavicular NBO received partial resection of the clavicle. Four patients with NBO received incisional/excisional biopsy. ^∗^Statistics and *P* values were for comparisons regarding cure rates between surgical and nonsurgical interventions in the BO and NBO patients, respectively.

## Data Availability

The data used to support the findings of this study are available from the corresponding author upon request.

## Abbreviations

BO: Bacterial osteomyelitis
NBO: Nonbacterial osteitis
OM: Osteomyelitis
SA: Septic arthritis
CRMO: Chronic recurrent multifocal osteomyelitis
SAPHO: Synovitis-acne-pustulosis-hyperostosis-osteitis
WBC: White blood cell count
ESR: Erythrocyte sedimentation rate
CRP: C-reactive protein
SPSS: Statistical Package for the Social Sciences
SD: Standard deviation
IQR: Interquartile range
CT: Computed tomography
MRI: Magnetic resonance imaging
BS: Bone scintigraphy
WB-MRI: Whole-body MRI.

## References

[1] Piazza C., Magnoni L., Nicolai P. (2006). Clavicular osteomyelitis: a rare complication after surgery for head and neck cancer. *European Archives of Oto-Rhino-Laryngology*.

[2] Yousef A., Pace A., Livesley P. (2006). Chronic haematogenous Pseudomonas aeruginosa osteomyelitis of the clavicle, a case report and review of the literature. *European Journal of Pediatrics*.

[3] Balakrishnan C., Vashi C., Jackson O., Hess J. (2008). Post-traumatic osteomyelitis of the clavicle: a case report and review of literature. *The Canadian Journal of Plastic Surgery*.

[4] Martins K., Lahiri A., Pupelis G. (2011). Surgical treatment of neurogenic thoracic outlet syndrome secondary to the clavicle haematogenic subacute osteomyelitis. *Journal of Hand Surgery*.

[5] Metsemakers W. J., Kuehl R., Moriarty T. F. (2018). Infection after fracture fixation: current surgical and microbiological concepts. *Injury*.

[6] Jiang N., Ma Y.-f., Jiang Y. (2015). Clinical characteristics and treatment of extremity chronic osteomyelitis in Southern China: a retrospective analysis of 394 consecutive patients. *Medicine*.

[7] Kremers H. M., Nwojo M. E., Ransom J. E., Wood-Wentz C. M., Melton III L. J., Huddleston III P. M. (2015). Trends in the epidemiology of osteomyelitis: a population-based study, 1969 to 2009. *The Journal of Bone and Joint Surgery*.

[8] Ross J. J. (2017). Septic arthritis of native joints. *Infectious Disease Clinics of North America*.

[9] Grote V., Silier C. C. G., Voit A. M., Jansson A. F. (2017). Bacterial osteomyelitis or nonbacterial osteitis in children: a study involving the German surveillance unit for rare diseases in childhood. *The Pediatric Infectious Disease Journal*.

[10] Hofmann S. R., Kapplusch F., Girschick H. J. (2017). Chronic recurrent multifocal osteomyelitis (CRMO): presentation, pathogenesis, and treatment. *Current Osteoporosis Reports*.

[11] Giedion A., Holthusen W., Masel L. F., Vischer D. (1972). Subacute and chronic symmetrical osteomyelitis. *Annales de Radiologie (Paris)*.

[12] Trobs R., Moritz R., Buhligen U. (1999). Changing pattern of osteomyelitis in infants and children. *Pediatric Surgery International*.

[13] Taddio A., Zennaro F., Pastore S., Cimaz R. (2017). An update on the pathogenesis and treatment of chronic recurrent multifocal osteomyelitis in children. *Pediatric Drugs*.

[14] Hu W. R., Yao Z. L., Yu B., Jiang N. (2019). Clinical characteristics and treatment of clavicular osteomyelitis: a systematic review with pooled analysis of 294 reported cases. *Journal of Shoulder and Elbow Surgery*.

[15] Zhang S., Li C., Shi X. (2018). Letters to the editor: refractory SAPHO syndrome with positive Th17-related pathway immunohistochemistry staining. *European Journal of Inflammation*.

[16] Yoshioka K., Nagata H., Nakamura Y. (1997). Pyogenic clavicular osteomyelitis associated with disseminated intravascular coagulation and acute renal failure in a patient with non-insulin-dependent diabetes mellitus. *Internal Medicine*.

[17] Wong L. S., Peh W. C. (2003). A 7-year-old boy with left sternoclavicular swelling. *American Journal of Orthopedics (Belle Mead, N.J.)*.

[18] Winslow C. P., Meyers A. D. (1998). Clavicular osteomyelitis as a complication of head and neck surgery. *Annals of Otology, Rhinology & Laryngology*.

[19] Winkler S., Dai L., Hauck F., Dinger J., Pessler F. (2012). Primary osteomyelitis of the clavicle in the newborn period. *Pediatric Infectious Disease Journal*.

[20] Wessel R. N., Schaap G. R. (2007). Outcome of total claviculectomy in six cases. *Journal of Shoulder and Elbow Surgery*.

[21] Weiner S. N., Levy M., Bernstein R., Morehouse H. (1984). Condensing osteitis of the clavicle. A case report. *The Journal of Bone and Joint Surgery. American Volume*.

[22] Watanakunakorn C. (1986). Serratia marcescens osteomyelitis of the clavicle and sternoclavicular arthritis complicating infected indwelling subclavian vein catheter. *The American Journal of Medicine*.

[23] Wang L., Li L.-F. (2017). Difficult clinical management of antituberculosis DRESS syndrome complicated by MRSA infection. *Medicine*.

[24] Walls T., Bate J., Moshal K. (2006). Vertebral collapse in an 8-year-old girl. *Journal of Paediatrics and Child Health*.

[25] Vierboom M. A., Steinberg J. D., Mooyaart E. L., van Rijswijk M. H. (1992). Condensing osteitis of the clavicle: magnetic resonance imaging as an adjunct method for differential diagnosis. *Annals of the Rheumatic Diseases*.

[26] van Holsbeeck M., van Melkebeke J., Dequeker J., Pennes D. R. (1992). Radiographic findings of spontaneous subluxation of the sternoclavicular joint. *Clinical Rheumatology*.

[27] Valerio P. G., Harmsen P. (1995). Osteomyelitis as a complication of perinatal fracture of the clavicle. *European Journal of Pediatrics*.

[28] Usman F., Bajwa A., Shujaat A., Cury J. (2011). Retrosternal abscess after trigger point injections in a pregnant woman: a case report. *Journal of Medical Case Reports*.

[29] Tingley R., Jadavji T., Boag G., Kiefer G. N., Trevenen C., Coppes M. J. (2001). Chronic recurrent multifocal osteomyelitis: a rare disorder presenting as multifocal bone lesions. *Medical and Pediatric Oncology*.

[30] Tickell K. D., Banim R., Kustos I. (2013). Salmonella sternoclavicular osteomyelitis in a patient with Crohn's disease. *BMJ Case Reports*.

[31] Thaddeus Chika A., Emeka O. M. (2016). Whole clavicle sequestration from chronic osteomyelitis in a 10 year old boy: a case report and review of the literature. *Annals of Medicine and Surgery*.

[32] Suranigi S. M., Joshi M., Deniese P. N., Rangasamy K., Najimudeen S., Gnanadoss J. J. (2016). Chronic osteomyelitis of clavicle in a neonate: report of morbid complication of adjoining MRSA abscess. *Case Reports in Pediatrics*.

[33] Sugase T., Akimoto T., Kanazawa H., Kotoda A., Nagata D. (2017). Sternocostoclavicular hyperostosis: an insufficiently recognized clinical entity. *Clinical Medicine Insights: Arthritis and Musculoskeletal Disorders*.

[34] Studemeister A. E., Dreyfuss B. J. (1990). Haematogenous clavicular osteomyelitis caused by Bacteroides fragilis. *Annals of the Rheumatic Diseases*.

[35] Strauss E. J., Kaplan K. M., Paksima N., Bosco J. A. (2008). Treatment of an open infected type IIB distal clavicle fracture: case report and review of the literature. *Bulletin of the NYU Hospital for Joint Diseases*.

[36] Stewart C. A., Siegel M. E., King D., Moser L. (1988). Radionuclide and radiographic demonstration of condensing osteitis of the clavicle. *Clinical Nuclear Medicine*.

[37] Sng K. K., Chan B. K., Chakrabarti A. J., Bell S. N., Low C. O. (2004). Condensing osteitis of the medial clavicle--an intermediate-term follow-up. *Annals Academy of Medicine Singapore*.

[38] Smith J., Yuppa F., Watson R. C. (1988). Primary tumors and tumor-like lesions of the clavicle. *Skeletal Radiology*.

[39] Sidhu G., Andrews G., Forster B., Keogh C. (2003). Residents' corner. Answer to case of the month #89. Chronic recurrent multifocal osteomyelitis as a presentation of SAPHO syndrome. *Canadian Association of Radiologists Journal*.

[40] Shodo R., Sato Y., Ota H., Horii A. (2017). Clavicle fracture with osteomyelitis after neck dissection and post-operative radiotherapy: case report. *Journal of Laryngology and Otology*.

[41] Sharma C., Chow B. (2017). A case of atypical synovitis-acne-pustulosis-hyperostosis-osteitis (SAPHO) syndrome presenting with osteomyelitis of the clavicle. *Wisconsin Medical Journal*.

[42] Saglam F., Saglam S., Gulabi D., Eceviz E., Elmali N., Yilmaz M. (2014). Bilateral clavicle osteomyelitis: a case report. *International Journal of Surgery Case Reports*.

[43] Rotbart H. A., Gelfand W. M., Glode M. P. (1984). Kingella kingae osteomyelitis of the clavicle. *Journal of Pediatric Orthopaedics*.

[44] Rosenfeld L. E. (1985). Osteomyelitis of the first rib presenting as a cold abscess nine months after subclavian venous catheterization. *Pacing and Clinical Electrophysiology*.

[45] Roos E., Maas M., Breugem S. J. M., Schaap G. R., Bramer J. A. M. (2015). Nonbacterial osteitis of the clavicle: longitudinal imaging series from initial diagnosis to clinical improvement. *Case Reports in Rheumatology*.

[46] Reith J. D., Bauer T. W., Schils J. P. (1996). Osseous manifestations of SAPHO synovitis, acne, pustulosis, hyperostosis, osteitis syndrome. *The American Journal of Surgical Pathology*.

[47] Rasool M. N., Govender S. (1991). Infections of the clavicle in children. *Clinical Orthopaedics and Related Research*.

[48] Quinn S. F., Oshman D. (1985). Case report 298. Osteomyelitis of the left clavicle due to Serratia marcescens. *Skeletal Radiology*.

[49] Prakash J., Aggarwal S., Mehtani A. (2014). Primary tuberculosis of the clavicle. *Orthopedics*.

[50] Petrovic I., Davila S., Premuzic I., Zdunic N., Trotic R., Prutki M. (2004). Long-term outcomes of clavicular pseudoarthrosis therapy. *Journal of Surgical Research*.

[51] Pelkonen P., Ryoppy S., Jaaskelainen J., Rapola J., Repo H., Kaitila I. (1988). Chronic osteomyelitislike disease with negative bacterial cultures. *Archives of Pediatrics & Adolescent Medicine*.

[52] Peces R., Diaz-Corte C., Baltar J., Gago E., Alvarez-Grande J. (1999). A desperate case of failing vascular access - management of superior vena cava thrombosis, recurrent bacteraemia, and acute clavicular osteomyelitis. *Nephrology Dialysis Transplantation*.

[53] Patel R., Naik S., Melnikau B. (2009). Clavicular tuberculosis following trivial trauma. *Southern Medical Journal*.

[54] Pan K. L., Chan W. H., Ong G. B., Zulqarnaen M., Norlida D. K. (2012). Non-bacterial chronic recurrent osteomyelitis of the clavicle. *Malaysian Orthopaedic Journal*.

[55] Pal C. P., Kumar H., Kumar S., Hussain A. (2016). Tubercular osteomyelitis of the lateral-third of the clavicle. *BMJ Case Reports*.

[56] Nurre L. D., Rabalais G. P., Callen J. P. (1999). Neutrophilic dermatosis‐associated sterile chronic multifocal osteomyelitis in pediatric patients: case report and review. *Pediatric Dermatology*.

[57] Noonan P. T., Stanley M. D., Sartoris D. J., Resnick D. (1998). Condensing osteitis of the clavicle in a man. *Skeletal Radiology*.

[58] Nishimura T., Kikuta S., Ishihara S., Nakayama S. (2017). Heart failure complicating with SAPHO syndrome. *BMJ Case Reports*.

[59] Mugnai G., Pesarini G., Vassanelli C. (2014). Osteomyelitis of the clavicle following to a pacemaker implantation. *EP Europace*.

[60] Mollan R. A., Craig B. F., Biggart J. D. (1984). Chronic sclerosing osteomyelitis. An unusual case. *The Journal of Bone and Joint Surgery. British volume*.

[61] Mikroulis D. A., Verettas D. A., Xarchas K. C., Lawal L. A., Kazakos K. J., Bougioukas G. J. (2008). Sternoclavicular joint septic arthritis and mediastinitis. A case report and review of the literature. *Archives of Orthopaedic and Trauma Surgery*.

[62] Meena U. K., Saibaba B., Behera P., Meena R. C. (2017). Sternoclavicular joint tuberculosis: a series of 9 cases. *Indian Journal of Tuberculosis*.

[63] Md Yusoff Z., Samadi E., Felix L. Y. S. (2018). Septic arthritis of a sternoclavicular joint caused by MRSA. *Malaysian Orthopaedic Journal*.

[64] Matzaroglou C., Velissaris D., Karageorgos A., Marangos M., Panagiotopoulos E., Karanikolas M. (2009). SAPHO syndrome diagnosis and treatment: report of five cases and review of the literature. *The Open Orthopaedics Journal*.

[65] Mangas-Loría C. A. J., Fuentes-Nucamendi M. A., Sánchez-Chávez A. F., Monreal-Chairez C., Ramos-Córdova C. (2018). Artritis septica acromioclavicular por Streptococcus agalactiae. Reporte de caso. *Revista Médica del Hospital General de México*.

[66] Lowden C. M., Walsh S. J. (1997). Acute staphylococcal osteomyelitis of the clavicle. *Journal of Pediatric Orthopaedics*.

[67] Lee J., Jeong C. H., Lee M. H. (2017). Emphysematous osteomyelitis due to Escherichia coli. *Infection & Chemotherapy*.

[68] Kyne S., Ntlholang O., Lucey R., O'Riordan D. (2017). Shoulder and clavicular pain: an insidious presentation of methicil-lin sensitive staphylococcus aureus infection. *Irish Journal of Medical Science*.

[69] Kumar T. K. J., Salim J., Shamsudeen T. J. (2018). Chronic recurrent multifocal osteomyelitis - a rare clinical presentation and review of literature. *Journal of Orthopaedic Case Reports*.

[70] Kruger G. D., Rock M. G., Munro T. G. (1987). Condensing osteitis of the clavicle. A review of the literature and report of three cases. *The Journal of Bone and Joint Surgery. American Volume*.

[71] Kravitz A. B. (1989). Osteomyelitis of the clavicle secondary to infected Hickman catheter. *Journal of Parenteral and Enteral Nutrition*.

[72] Kotilainen P. M., Laxen F. O., Manner I. K., Gullichsen R. E., Saario R. M. (1996). An aseptic inflammation of the clavicle in a patient with Crohn's disease. A potential manifestation of the SAPHO syndrome. *Scandinavian Journal of Rheumatology*.

[73] Kotilainen P., Gullichsen R. E., Saario R., Manner I., Kotilainen E. (1997). Aseptic spondylitis as the initial manifestation of the SAPHO syndrome. *European Spine Journal*.

[74] Klein B., Mittelman M., Katz R., Djaldetti M. (1983). Osteomyelitis of both clavicles as a complication of subclavian venipuncture. *Chest*.

[75] Khan M. A., Osborne N. J., Anaspure R., Ramanan A. V. (2013). Clavicular swelling-classic presentation of chronic non-bacterial osteomyelitis. *Archives of Disease in Childhood*.

[76] Kennedy M. T., Murphy T., Murphy M., Laffan E., Connolly P. (2012). Whole body MRI in the diagnosis of chronic recurrent multifocal osteomyelitis. *Orthopaedics & Traumatology: Surgery & Research*.

[77] Kambhampati G., Asmar A., Pakkivenkata U., Ather I. S., Ejaz A. A. (2011). Anaerobic clavicular osteomyelitis following colonoscopy in a hemodialysis patient. *Clinical and Experimental Nephrology*.

[78] Jurik A. G., Moller B. N. (1987). Chronic sclerosing osteomyelitis of the clavicle. A manifestation of chronic recurrent multifocal osteomyelitis. *Archives of Orthopaedic and Trauma Surgery*.

[79] Jurik A. G., Moller B. N. (1986). Inflammatory hyperostosis and sclerosis of the clavicle. *Skeletal Radiology*.

[80] Jurik A. G., Egund N. (1997). MRI in chronic recurrent multifocal osteomyelitis. *Skeletal Radiology*.

[81] Judich A., Haik J., Rosin D., Kuriansky J., Zwas S. T., Ayalon A. (1998). Osteomyelitis of the clavicle after subclavian vein catheterization. *Journal of Parenteral and Enteral Nutrition*.

[82] Jones M. W., Carty H., Taylor J. F., Ibrahim S. K. (1990). Condensing osteitis of the clavicle: does it exist?. *The Journal of Bone and Joint Surgery. British Volume*.

[83] Jilkine K., Boyd T. (2018). A 38-year-old man with isolated sternoclavicular joint swelling. *Journal of Rheumatology*.

[84] Jibri Z., Sah M., Mansour R. (2012). Chronic recurrent multifocal osteomyelitis mimicking osteoid osteoma. *Journal Belge de Radiologie - Belgisch Tijdschrift voor Radiologi*.

[85] Imran M. B., Othman S. (2012). Bilateral condensing osteitis of clavicles: differential diagnosis of an unusual case. *Rheumatology International*.

[86] Hunter D., Moran J. F., Venezio F. R. (1983). Osteomyelitis of the clavicle after Swan-Ganz catheterization. *Archives of Internal Medicine*.

[87] Hsu C. Y., Frassica F., McFarland E. G. (1998). Condensing osteitis of the clavicle: case report and review of the literature. *The American Journal of Orthopedics (Belle Mead, N.J.)*.

[88] Harden S. P., Argent J. D., Blaquiere R. M. (2004). Painful sclerosis of the medial end of the clavicle. *Clinical Radiology*.

[89] Guglielmi G., Cascavilla A., Scalzo G., Salaffi F., Grassi W. (2009). Imaging of sternocostoclavicular joint in spondyloarthropaties and other rheumatic conditions. *Clinical and Experimental Rheumatology*.

[90] Griffith J. F., Kumta S. M., Chow L. T. C., King A. D., Leung P. C. (1998). Sclerosis and swelling of the clavicle in a 44-year-old woman. *Clinical Orthopaedics and Related Research*.

[91] Girschick H. J., Krauspe R., Tschammler A., Huppertz H. I. (1998). Chronic recurrent osteomyelitis with clavicular involvement in children: diagnostic value of different imaging techniques and therapy with non-steroidal anti-inflammatory drugs. *European Journal of Pediatrics*.

[92] Gikas P. D., Islam L., Aston W. (2009). Nonbacterial osteitis: a clinical, histopathological, and imaging study with a proposal for protocol-based management of patients with this diagnosis. *Journal of Orthopaedic Science*.

[93] Gicchino M. F., Diplomatico M., Granato C. (2018). Chronic recurrent multifocal osteomyelitis: a case report. *Italian Journal of Pediatrics*.

[94] Ghate S., Thabet A. M., Gosey G. M., Southern E. P., Bégué R. E., King A. G. (2016). Primary osteomyelitis of the clavicle in children. *Orthopedics*.

[95] Gerscovich E. O., Greenspan A. (1994). Osteomyelitis of the clavicle: clinical, radiologic, and bacteriologic findings in ten patients. *Skeletal Radiology*.

[96] Garcia S., Combalia A., Segur J. M., Llovera A. J. (1999). Osteomyelitis of the clavicle. A case report. *Acta Orthopaedica Belgica*.

[97] Franquet T., Lecumberri F., Rivas A., Inaraja L., Idoate M. A. (1985). Condensing osteitis of the clavicle. Report of two new cases. *Skeletal Radiology*.

[98] Franekova L., Pasek P. (2017). Giant manubrium sterni by patient with sapho syndrome: case report. *Osteoporosis International*.

[99] Falip C., Alison M., Boutry N. (2013). Chronic recurrent multifocal osteomyelitis (CRMO): a longitudinal case series review. *Pediatric Radiology*.

[100] Erhardt E., Harangi F. (1997). Two cases of musculoskeletal syndrome associated with acne. *Pediatric Dermatology*.

[101] Epperla N., Kattamanchi S., Fritsche T. R. (2015). Appearances are deceptive: Staphylococcus superinfection of clavicular tuberculous osteomyelitis. *Clinical Medicine & Research*.

[102] Ely G. M. (1999). Septic arthritis of the sternoclavicular joint and osteomyelitis of the proximal clavicle caused by prevotella melaninogenicus: a case with several features delaying diagnosis. *Journal of Clinical Rheumatology*.

[103] Eftekhari F., Jaffe N., Schwegel D., Ayala A. (1989). Inflammatory metachronous hyperostosis of the clavicle and femur in children. Report of two cases, one with long-term follow-up. *Skeletal Radiology*.

[104] Duro J. C., Estrada P., Ribas D., Bartrons S., Rotes-Querol J. (1981). Condensing osteitis of the clavicle. *Arthritis & Rheumatology*.

[105] Dugg P., Shivhare P., Mittal S., Singh H., Tiwari P., Sharma A. (2013). Clavicular osteomyelitis: a rare presentation of extra pulmonary tuberculosis. *Journal of Surgical Case Reports*.

[106] Donovan R. M., Shah K. J. (1982). Unusual sites of acute osteomyelitis in childhood. *Clinical Radiology*.

[107] Docquier P. L., Malghem J., Mousny M., Rombouts J. J. (2006). Chronic osteomyelitis of clavicle as primary manifestation of SAPHO syndrome in adolescents: report of four cases and long-term evolution. *Joint, Bone, Spine*.

[108] De Lord D. A., Thomas A. M., Salisbury J., Pitt P. I. (1996). A case of chronicEscherichia coliosteomyelitis of the clavicle. *British Journal of Rheumatology*.

[109] de Kort J. G. J. L., Robben S. G. F., Schrander J. J. P., van Rhijn L. W. (2006). Multifocal osteomyelitis in a child: a rare manifestation of cat scratch disease: a case report and systematic review of the literature. *Journal of Pediatric Orthopaedics. Part B*.

[110] de Belder K. R. (1985). Excision of the clavicle. A review of the nineteenth-century literature. *The Journal of Bone and Joint Surgery. British Volume*.

[111] Damodaran A., Rohit A., Abraham G., Nair S., Yuvaraj A. (2014). Case report: rare occurrence of Pseudomonas aeruginosa osteomyelitis of the right clavicle in a patient with IgA nephropathy. *F1000Research*.

[112] Cone R. O., Resnick D., Goergen T. G., Robinson C., Vint V., Haghighi P. (1983). Condensing osteitis of the clavicle. *American Journal of Roentgenology*.

[113] Clement N. D., Nicol G., Porter D. E. (2014). Nontraumatic lesions of the clavicle in a paediatric population: incidence and management. *International Scholarly Research Notices*.

[114] Chun J. M., Kim J. S., Jung H. J. (2012). Resection arthroplasty for septic arthritis of the sternoclavicular joint. *Journal of Shoulder and Elbow Surgery*.

[115] Chrysochoou E.-A., Antachopoulos C., Badekas K., Roilides E. (2016). A rare case of clavicle osteomyelitis in a child and literature review. *Case Reports in Pediatrics*.

[116] Choke A., Yang Y. O., Koh J. S. B., Howe T. S., Tan B.-K. (2018). Restoring a functional and mobile shoulder following reconstruction of the sternoclavicular joint with a free vascularized fibular flap. *JPRAS Open*.

[117] Carpenter M. R., Vassallo M., Bourke W., Sell P. J., Pohl J. E., Scriven A. J. (1997). Shoulder pain and pyrexia following subclavian line insertion. *Postgraduate Medical Journal*.

[118] Carpenter E., Jackson M. A., Friesen C. A., Scarbrough M., Roberts C. C. (2004). Crohns-associated chronic recurrent multifocal osteomyelitis responsive to infliximab. *The Journal of Pediatrics*.

[119] Callcott F., Gordon L., Schabel S. I., Friedman R. (1988). Indium-111 WBC imaging--false-positive in a simple fracture. *Journal of Nuclear Medicine*.

[120] Burns P., Sheahan P., Doody J., Kinsella J. (2008). Clavicular osteomyelitis: a rare complication of head and neck cancer surgery. *Head & Neck*.

[121] Boruah D. K., Prakash A., Gogoi B. B., Sanyal S., Sarkar C., Bora S. (2018). Tubercular osteomyelitis of clavicle: a rare clinico-radiological diagnostic dilemma and master mimicker. *Journal of Clinical and Diagnostic Research*.

[122] Bleckwenn M., Sommer B., Weckbecker K. (2014). Chronic recurrent multifocal osteomyelitis manifested as painful clavicular swelling: a case report. *BMC Research Notes*.

[123] Bjorksten B., Boquist L. (1980). Histopathological aspects of chronic recurrent multifocal osteomyelitis. *The Journal of Bone and Joint Surgery. British volume*.

[124] Belfiore N., Caporali R., Borroni G., Montecucco C. (1997). Anterior chest wall arthritis and osteitis associated with Sneddon-Wilkinson disease. *Clinical and Experimental Rheumatology*.

[125] Barrani M., Massei F., Scaglione M. (2015). Unusual onset of a case of chronic recurrent multifocal osteomyelitis. *Pediatric Rheumatology Online Journal*.

[126] Baratz M., Appleby D., Fu F. H. (1985). Life-threatening clavicular osteomyelitis in two debilitated patients. *Orthopedics*.

[127] Azouz E. M., Jurik A. G., Bernard C. (1998). Sternocostoclavicular hyperostosis in children: a report of eight cases. *American Journal of Roentgenology*.

[128] Appell R. G., Oppermann H. C., Becker W., Kratzat R., Brandeis W. E., Willich E. (1983). Condensing osteitis of the clavicle in childhood: a rare sclerotic bone lesion. Review of literature and report of seven patients. *Pediatric Radiology*.

[129] Andreacchio A., Marengo L., Canavese F. (2016). Condensing osteitis of the clavicle in children. *World Journal of Orthopedics*.

[130] Altiok I. B., Tokmak M., Akman T., Alkan B., Cosar M. (2014). Condensing osteitis of the clavicle in a man: any relationship with tooth decay?. *Journal of Pakistan Medical Association*.

[131] Ali S., Arnold A., Lee S., Lucas M., Rueter K. (2018). P24: anaphylaxis to methotrexate in a child with chronic recurrent multifocal osteomyelitis. *Internal Medicine Journal*.

[132] Al-Fifi S. H., Al-Qahtani S. M., Al-Binali A. M., Annobil S. H. (2002). An unusual complication of sternal and clavicle osteomyelitis in a child with sickle cell disease. *Saudi Medical Journal*.

[133] Alessi D. M., Sercarz J. A., Calcaterra T. C. (1988). Osteomyelitis of the clavicle. *Archives of Otolaryngology - Head and Neck Surgery*.

[134] Akhtar M. N., Agarwal S., Athar R. (2015). Clinico-radiological approach to a rare case of early clavicle tuberculosis: a case discussion based review of differential diagnosis. *Journal of Clinical and Diagnostic Research*.

[135] Aggarwal A. N., Dhammi I. K., Singh A. P., Kumar S., Goyal M. K. (2009). Tubercular osteomyelitis of the clavicle: a report of four cases. *Journal of Orthopaedic Surgery*.

[136] Agarwal A., Maheshwari R. (2014). Tubercular osteomyelitis clavicle: a case report. *Journal of Orthopaedic Case Reports*.

[137] Acus R. W., Bell R. H., Fisher D. L. (1995). Proximal clavicle excision: an analysis of results. *Journal of Shoulder and Elbow Surgery*.

[138] Abril J. C., Castillo F., Loewinsonh A. F., Rivas C., Bernacer M. (1994). Chronic recurrent multifocal osteomyelitis after acute lymphoblastic leukaemia. *International Orthopaedics*.

[139] Kaiser D., Bolt I., Hofer M. (2015). Chronic nonbacterial osteomyelitis in children: a retrospective multicenter study. *Pediatric Rheumatology Online Journal*.

[140] Bhat C. S., Anderson C., Harbinson A. (2018). Chronic non bacterial osteitis- a multicentre study. *Pediatric Rheumatology Online Journal*.

[141] Jansson A., Renner E. D., Ramser J. (2007). Classification of non-bacterial osteitis: retrospective study of clinical, immunological and genetic aspects in 89 patients. *Rheumatology (Oxford)*.

[142] Metsemakers W. J., Morgenstern M., McNally M. A. (2018). Fracture-related infection: a consensus on definition from an international expert group. *Injury*.

[143] Jiang N., Zhao X. Q., Wang L., Lin Q. R., Hu Y. J., Yu B. (2020). Single-stage debridement with implantation of antibiotic-loaded calcium sulphate in 34 cases of localized calcaneal osteomyelitis. *Acta Orthopaedica*.

